# How the government intervention affects the distribution of physicians in Turkey between 1965 and 2000

**DOI:** 10.1186/s12939-014-0131-1

**Published:** 2015-01-08

**Authors:** Erdinç Ünal

**Affiliations:** Faculty of Economics and Administrative Sciences, Okan University, İstanbul, Turkey

**Keywords:** Distribution of physicians, Government intervention on health, Compulsory service for physicians

## Abstract

**Introduction:**

One of the main weaknesses of the health system in Turkey is the uneven distribution of physicians. The diversity among geographical districts was huge in the beginning of the 1960s. After the 1980s, the implementation of a two-year compulsory service for newly graduated physicians is an interesting and specific experience for all countries. The aim of this study is to analyse the distribution of physicians, GPs and specialists between the years 1965-2000 and the efficiency of the strict 15 year government intervention (1981-1995).

**Methods:**

The data used in this study includes the published data by the Ministry of Health and The State Institute of Statistics between the years 1965–2000. Covering 35 years for total physicians, GPs and specialists, Gini coefficients are calculated so as to observe the change in the distribution. In order to measure the efficiency of government intervention, Gini index belonging to the previous 15 years (first period-1965 to 1980) and the last 15 years (second period) of 1981 when the compulsory service was enacted is also analysed including the statistical tests.

**Results:**

In 1965, the Gini for total physician is quite high (0.47), and in 2000 it decreases considerably (0.20). In 1965, the Gini for GPs and the Gini for specialists is 0.44 and 0.52, respectively and in 2000 these values decrease to 0.13 and 0.28, respectively. It is observed that, with this government intervention, the level of diversity has decreased dramatically up to 2000. Regarding to regression, the rate of decrease in Gini index in the second period is higher for the GPs than that of the specialists.

**Conclusion:**

The inequalities in the distribution between GPs and specialists are significantly different; inequality of specialist distribution is higher than the GP. The improvement of the inequality in the physician distribution produced by the market mechanism shows a long period when it is left to its own devices. It is seen that the compulsory service policy is efficient since the physician distribution has improved significantly. The government intervention provides a faster improvement in the GP distribution.

## Introduction

The unequal distribution of physicians is a fact seen almost all over the world. The distribution of human resources in health care has been recognised as one of the most important issues for the evaluation of persistent inequities. This problem is not peculiar to Turkey and could be seen throughout the world as well [1-4]. Any differences in the distribution of health care personnel density are seen in various regions of all countries. But these differences are also seen in the cities of each region and, moreover, they are also encountered in the surrounding areas and suburbs of each metropolis [5].

The inequality in distribution of physicians was generally higher than other health human resources [6]. To provide a fair distribution of physicians between developed urban areas and underdeveloped rural areas has been a continuous effort of the decision-makers of health policy and practitioners of national health policy in almost all countries. Planning the geographical distribution of physicians has been one of the most important policy implications. Similar to many countries, the problem of arranging the distribution of physicians with the aim of meeting the needs of national health organisation and the public demand have always been on the agenda of the Turkish governments.

### Health services and market failures

Health services used to advance out of the market mechanism in many ways throughout the world. The motivations and mechanism of the market cannot provide a socially efficient production and a fair distribution of the health services. This means the failure of efficiency and equity, both of which are expected from an every economic activity, and the situation that arises when these two concepts do not happen as expected is the basic subject of market failures.

Due to the increase in the demand for healthcare in big cities, employing a greater number of physicians is an expected case. Demand is not the sole reason of physician density in big and developed cities/regions.^a^ The factors affecting the physician distribution are divided into four categories: (1) supportive facilities; (2) socioeconomic and demographic characteristics of an area; (3) socio-cultural considerations; and (4) need for medical services [7,8].

The market failure argument about the physician distribution is related to the intensive distribution of physicians in more advantaged areas. Even though the regions and cities that could be called more advantaged than others reach a saturation point in regard to the number of physicians, the market failure continues to exist. Cities and developed regions in developing countries especially continue to absorb newly graduated physicians due to an inadequate supply of physicians. Another factor valid for both developing and developed countries is the increasing demand for new medical services in developed regions and cities. Of course, physicians don’t have the ability to create demand unlimitedly, but they could face a loss of income to a certain extent. Even in these conditions, they prefer living in large cities and socioeconomically developed urban areas.

“The quantitative evidence supporting the case for market failure usually takes two forms: (1) At each point in time, physician/population ratios in nonmetropolitan counties are markedly lower than those in metropolitan counties. (2) Over time, physician/population ratios in small towns or counties have risen more slowly than those in metropolitan areas” [9].

In their studies, Newhouse et al. [10], consider the total population as a critical factor in the distribution of physicians since they prefer the areas with a higher population to have sufficient demand. Besides, they not only seek to maximize their profits but also to increase the quality of their social life profile, non-cash benefits and access to the medical facilities [3,8].

The distribution of health labour power in the population and geography is an important element in terms of reproducibility and availability of health services. Physician supply is the most important element for equitability in access to medical care. Intervention of the government appears where there is an unbalanced physician distribution. Taking measures in regard to a balanced physician distribution will improve the allocation of human resources in health system [11]. On the other hand, the medical staffs, especially physicians, prefer living in socially and economically developed cities, regions and metropolitan areas in the country [2,12].

The market mechanism is insufficient to provide an optimum geographical distribution, leading to a great failure. In such cases, it is possible to provide a better distribution of physicians through utilisation of appropriate public policies. This was one of the most important problems in the health systems of the leader countries of free market mechanisms such as US and Britain in the 1960s. Even today, it is still possible to see this problem but to a lesser degree due to the effects of applied interventional policies [1,4,13].

Therefore, the distribution of physicians has always been subject to governmental intervention at universal level. The provision of equal access to health care providers in all regions as far as possible must be one of the targets for the health system of a country. The governments are developing two main policies in this field: The first one is to increase the number of physicians and the second one is to improve the geographical distribution of physicians with several arrangements.

### Geographical distribution of physicians in Turkey

The level of regional inequalities in the geographic distribution of physicians was very high in the early years of the Republic of Turkey. Inasmuch as there was a shortage of physicians throughout the country which was in the beginning phase of the socioeconomic development, the results of unrestrained distribution of physicians did not pose any problem for the government. Together with the increase in the number of physicians, this trend continued. However, the inequalities in the distribution of physicians and the problem of physician shortage in rural areas were often put on the agenda of the politicians by the people living in rural and underdeveloped areas which were in need of health service. Despite the political efforts of the governments that generally increase in the run-up to elections, a well-balanced distribution of physicians could not be achieved; on the contrary, the law that was enacted to improve and regulate the distribution of physicians in the country and that included the compulsory service was considered to be valid as of August 1981. According to “The Law Regarding the Obligation of Civil Service for Some Medical Staff”, it became obligatory for the newly appointed general practitioners (GPs) and the specialists to do a two year compulsory public service. This law was in force for 15 years between 1981 and 1995.

A fair distribution of the physicians throughout the country was the main aim of this law in which the health authority (The Ministry of Health) determined where the newly appointed physicians would work. Thanks to this unique experience, Turkey set a prime example to all countries in the world in regard to what extent the distribution of physicians would be affected or changed by legal arrangements.

The study, in short, consists of the distribution of physicians in Turkey during 35 years that includes 15 years of strict government intervention and the comparison of periods before and during this intervention. As a correcting mechanism, was the legislation about the distribution of physicians efficient, and how? The aim of this study is to present the unique experience of Turkey through the scientific analysis method, which would be a guide for the legislators and political decision-makers.

## Materials and methods

In this study, the inequalities and the change in the distribution of physicians in Turkey between 1965 and 2000 are analysed. Besides, the periods before 1980 when the distribution of physicians was not governmentally regulated and after 1980 till 1995 when compulsory service law was applied strictly are comparatively examined. The years from 1965 to 1980 are labelled as the first period while the years from 1981 to 1995 are defined as the second period. To what extent the legal arrangement as a public intervention was successful in providing the even distribution that the market failed to do is assessed.

The data used in this study includes the published data by the Ministry of Health and The State Institute of Statistics between the years 1965 and 2000. There was a noticeable decrease in the effect of the regulation between the years 1995 and 2000 when the law was suspended. During this five-year period, the rate of decrease in Gini index apparently diminished. At the same time, the fact that the data was cut due to the change of regional definition by the Ministry of Health after 2000 has meant that the period after 1995 could not be included in the comparative analysis. In addition, the data regarding the population of regions for the term between 2000 and 2007 could not be obtained due to certain alterations in the census system of the Turkish Statistical Institute.

In Turkey, every physician who works in their own clinics or every hospital and clinic must inform the Ministry of Health about the place where they work. According to the legal regulations, the doctors cannot work outside of their region. Therefore, the data used in this study covers all the physicians in the country and they are categorised into two groups according to being specialist or not. In these analyses, the data on the distribution of physicians both for GPs and specialists is present.

**Figure 1:**
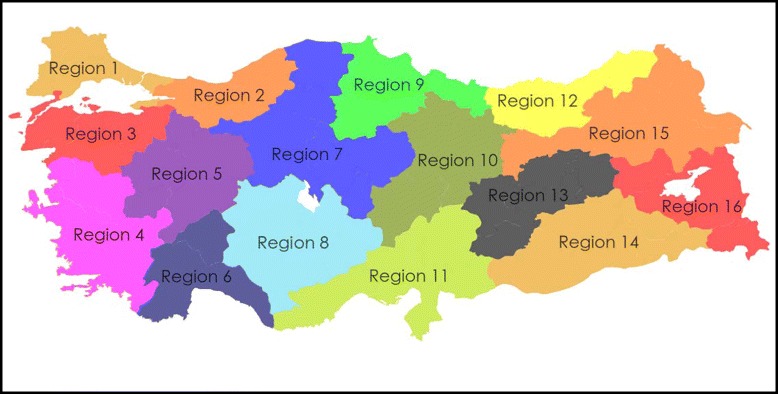
**Map of Turkey in respect to health regions determined by the Ministry of Health.**

Sixteen groups were defined according to the regional city groups that contain a few (generally 3–4) neighbour cities by Ministry of Health (Figure 1). In general, the initial groups cover cities with high population, or located in coasts and/or at the regional economic centres. They have approximately 2/3 of Turkey’s population. From top to down, the cities in the groups are getting smaller, more rural, underdeveloped and lower population.

The distribution of physicians is organised as the ratio of population to physician in every each 16 groups for 35 years at three different categories (total physicians, GPs and specialists). This measurement is a basic and simple indicator of the physician distribution. The other measurements of distribution or mal-distribution are Gini index, Atkinson index, Theil index, etc. The Gini index has been widely used to compare geographic distributions of physicians among regions or over time [5,14]. The inequality in the distribution of physicians is measured through using Gini coefficient indices and population to physician ratios in this study. The Gini coefficient is derived from the Lorenz curve of the plot of cumulative percentage of the population by socio-economic status and cumulative percentage of total income. The Gini coefficient is calculated as the ratio of the area between the Lorenz curve and the 45° line, to the whole area below the 45° line; a Gini coefficient of 0 reflects a perfectly equal society, and a Gini coefficient of 1 represents a perfectly unequal society [15,16]. The Brown formula is used for this purpose [17].

$$ \boldsymbol{G}=1-{\displaystyle \sum_{\boldsymbol{i}=0}^{\boldsymbol{k}-1}}\left\{{\boldsymbol{Y}}_{\boldsymbol{i}+1} + {\boldsymbol{Y}}_{\boldsymbol{i}}\right\}\ \left\{{\boldsymbol{X}}_{\boldsymbol{i}+1}\ \hbox{--}\ {\boldsymbol{X}}_{\boldsymbol{i}}\right\} $$

G: Gini coefficient

Yi: Cumulative proportion of the physicians (total, GP sor specialists) in the *ith* region

Xi: Cumulative proportion of the population variable in the *i*th region

k: total number of region

In the operationalised using of this formula, gini coefficents were derived from the Lorenz curve with plotting the region having the highest population per physician (starting from the worst to the best among the 16 regions), the corresponding cumulative population ratio of the region to the cumulative physician number of that region.

Covering 35 years for total physicians, GPs and specialists, Gini coefficients are calculated so as to observe the change in the distribution. While the physicians have a right to express their preferences in their work and settlement place before 1981 (first period), during the compulsory service legislation period (second period), the newly graduated physicians (both GPs and specialists) have to work for two years in the place which is already appointed by the Ministry of Health. Changes in the distribution of physicians between the first and the second period are compared. In order to measure the efficiency of government intervention, changes in Gini index for both periods are analysed including the statistical tests. The effect of independent variables (years) on dependent variable (Gini index) is diagnosed via multiple linear regression analysis using SPSS after preliminary regression assumptions are confirmed. The effect of law intervention is examined from two perspectives. The first one is between periods (pre-after 1981) and, second one is between GPs and specialists in the second period. The effect of group differentiation is analysed through Mann-Whitney *U* test due to limited number of observations that does not fit with normal distribution. Thus we tested whether the rates of decrease of the Gini coefficients for the GP and specialist were equal over the period from 1981 to 1995 using the Mann-Whitney *U* test.

## Results

### Trends in number of physicians and their geographic distribution

**Figure 2:**
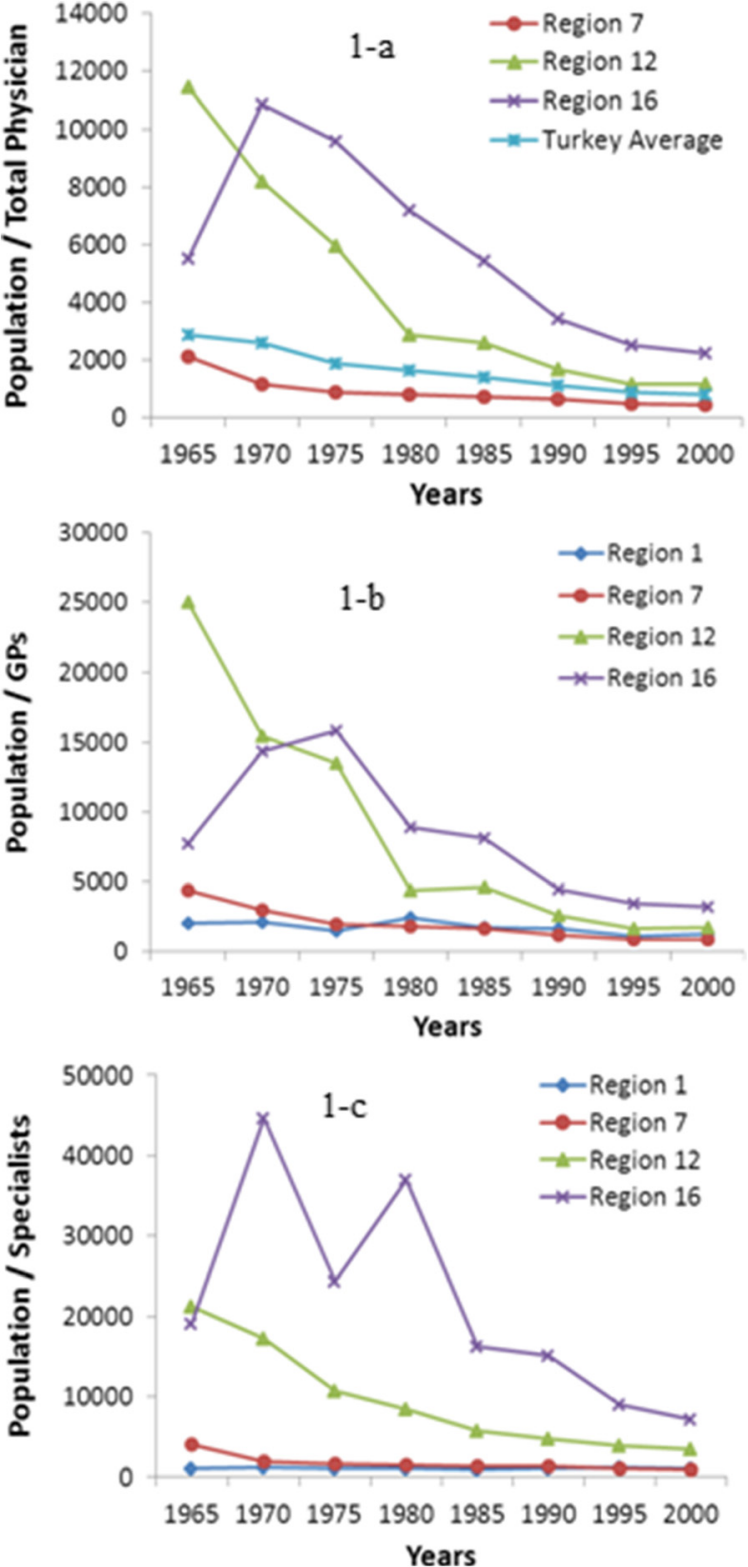
**Change on the ratios of population to physicians for total (a), GP’s (b) and (c) specialists in regions that have the most and least ratios of population to physicians.**

In 1965, the average population to physicians is 2881 in Turkey; the Region 1 has the best ratio with 675 and the Region 12 has the worst ratio with 11471 (approximately 17 times). The new student quotas and number of medical schools were increased in Turkey after 1980’s. While the number of physicians was significantly increasing, compulsory service law was levied at the same period to improve the distribution of physicians. Hence, the ratios of population to physicians (for total, GPs and specialists) decreased dramatically. In the year 2000, the average population to physicians is 792 in Turkey; the Region 7 has the best ratio with 445 and the Region 16 has the worst ratio with 2213 (approximately 5 times) (Table 1 and Figure 2).

**Table 1:** **Regional distribution of physicians in Turkey 1965-2000**

		**Years**	**1965**	**1970**	**1975**	**1980**	**1985**	**1990**	**1995**	**2000**
**Total population/total physician**			**2881**	**2572**	**1858**	**1642**	**1391**	**1115**	**890**	**792**
**Region 1**	**Total phys.**	Number of.	4654	5350	7959	8215	11403	13495	17551	19392
		Pop/Physc	675	728	607	700	608	629	574	590
	**Specialists**	Number of.	3138	3493	4721	5860	7328	8272	8646	10404
		Pop/Specia	1002	1115	1024	981	946	1027	1166	1099
	**GP's**	Number of.	1516	1857	3238	2367	4075	5223	8905	8988
		Pop/GP's	2073	2098	1493	2429	1701	1626	1132	1272
**Region 2**	**Total phys.**	Total Physc	337	374	643	681	1150	1671	2628	3335
		Pop/Physc	5264	5326	3481	3777	2524	1933	1343	1068
	**Specialists**	Number of.	211	238	425	415	661	827	1149	1477
		Pop/Specia	8408	8370	5266	6198	4392	3906	3072	2412
	**GP's**	Number of.	126	136	218	266	489	844	1479	1858
		Pop/GP's	14079	14647	10266	9669	5937	3827	2387	1917
**Region 3**	**Total phys.**	Total Physc	373	442	673	666	1490	2346	3157	4009
		Pop/Physc	4863	4430	3150	3593	1780	1283	1055	909
	**Specialists**	Number of.	282	316	425	455	887	1266	1459	1784
		Pop/Specia	6433	6196	4988	5259	2990	2377	2283	2043
	**GP's**	Number of.	91	126	247	211	603	1080	1698	2225
		Pop/GP's	19934	15540	8583	11341	4398	2786	1961	1638
**Region 4**	**Total phys.**	Total Physc	1281	1401	3011	3375	4776	6606	9253	11935
		Pop/Physc	2581	2625	1368	1367	1102	906	720	596
	**Specialists**	Number of.	802	933	1784	1846	2895	3266	3948	5434
		Pop/Specia	4122	3941	2308	2499	1819	1833	1687	1308
	**GP's**	Number of.	479	468	1227	1529	1881	3340	5305	6501
		Pop/GP's	6902	7857	3356	3017	2799	1793	1255	1094
**Region 5**	**Total phys.**	Total Physc	261	307	455	634	1027	1533	2223	2510
		Pop/Physc	6303	5824	4200	3207	2181	1581	1156	1069
	**Specialists**	Number of.	163	207	277	371	502	609	825	943
		Pop/Specia	10092	8638	6899	5480	4462	3980	3115	2845
	**GP's**	Number of.	98	100	178	263	525	924	1398	1567
		Pop/GP's	16786	17880	10736	7730	4267	2623	1838	1712
**Region 6**	**Total phys.**	Total Physc	185	195	248	537	736	1349	2369	3205
		Pop/Physc	5124	5579	4899	2484	2068	1351	894	770
	**Specialists**	Number of.	113	136	165	257	385	624	960	1372
		Pop/Specia	8389	8000	7364	5191	3953	2920	2205	1798
	**GP's**	Number of.	72	59	83	280	351	725	1409	1833
		Pop/GP's	13167	18441	14639	4764	4336	2513	1503	1346
**Region 7**	**Total phys.**	Total Physc	1503	3142	4932	5816	7069	8582	12125	14044
		Pop/Physc	2112	1165	866	785	722	631	465	445
	**Specialists**	Number of.	782	1927	2720	3247	3995	452	5714	6616
		Pop/Specia	4060	1899	1570	1406	1278	1336	986	948
	**GP's**	Number of.	721	1215	2212	2557	3074	4530	6411	7341
		Pop/GP's	4404	3012	1931	1786	1660	1195	879	851
**Region 8**	**Total phys.**	Total Physc	194	214	259	281	859	1535	2171	2634
		Pop/Physc	7655	7893	7282	7381	2711	1694	1292	1200
	**Specialists**	Number of.	114	152	163	129	426	612	719	900
		Pop/Specia	13026	11112	11571	16078	5467	4248	3903	3511
	**GP's**	Number of.	80	62	96	152	433	923	1452	1734
		Pop/GP's	18563	27242	19646	13645	5379	2817	1933	1822
**Region 9**	**Total phys.**	Total Physc	252	274	454	553	1088	1736	2512	3075
		Pop/Physc	9274	9201	5967	5264	2858	1855	1299	1068
	**Specialists**	Number of.	169	203	314	302	540	673	887	1236
		Pop/Specia	13828	12419	8627	9639	5759	4786	3680	2657
	**GP's**	Number of.	83	71	140	251	548	1063	1625	1839
		Pop/GP's	28157	35507	19350	11598	5675	3030	2009	1786
**Region 10**	**Total phys.**	Total Physc	192	224	332	611	1101	1810	2104	2639
		Pop/Physc	9047	8353	6078	3524	2103	1343	1187	999
	**Specialists**	Number of.	127	151	222	276	513	754	709	882
		Pop/Specia	13677	12391	9090	7801	4513	3223	3523	2990
	**GP's**	Number of.	65	73	110	335	588	1056	1395	1757
		Pop/GP's	26723	25630	18346	6427	3937	2301	1791	1501
**Region 11**	**Total phys.**	Total Physc	606	625	961	1352	2478	3097	4389	6786
		Pop/Physc	4736	5365	4222	3501	2247	2049	1518	1116
	**Specialists**	Number of.	385	431	633	744	1443	1514	1788	2746
		Pop/Specia	7455	7780	6410	6362	3859	4191	3727	2758
	**GP's**	Number of.	221	194	328	608	1035	1583	2553	3764
		Pop/GP's	12986	17284	12369	7785	5381	4008	2610	2012
**Region 12**	**Total phys.**	Total Physc	155	237	342	723	846	1268	1742	2064
		Pop/Physc	11471	8165	5965	2873	2569	1682	1167	1131
	**Specialists**	Number of.	84	112	191	245	376	449	531	689
		Pop/Specia	21167	17277	10681	8478	5779	4751	3829	3388
	**GP's**	Number of.	71	125	151	478	470	819	1211	1375
		Pop/GP's	25042	15480	13510	4345	4623	2604	1679	1698
**Region 13**	**Total phys.**	Total Physc	147	220	282	330	564	963	1677	2227
		Pop/Physc	9163	6941	6082	5461	3500	2179	1302	1071
	**Specialists**	Number of.	57	106	162	118	229	294	479	656
		Pop/Specia	23632	14406	10586	15271	8620	7136	4557	3636
	**GP's**	Number of.	90	114	120	212	335	669	1198	1571
		Pop/GP's	14967	13395	14292	8500	5893	3136	1822	1518
**Region 14**	**Total phys.**	Total Physc	245	310	43	383	745	1707	1780	2240
		Pop/Physc	6486	6106	4451	6243	3901	2053	2275	2030
	**Specialists**	Number of.	81	179	220	120	243	678	480	687
		Pop/Specia	19617	10575	9773	19925	11959	5168	8435	6620
	**GP's**	Number of.	164	131	263	263	502	1029	1297	1553
		Pop/GP's	9689	14450	8175	9091	5789	3405	3122	2929
**Region 15**	**Total phys.**	Total Physc	382	450	576	647	843	1066	1471	1832
		Pop/Physc	4555	4247	3590	3326	2728	2108	1446	1313
	**Specialists**	Number of.	112	215	234	268	371	314	438	549
		Pop/Specia	15536	8888	8838	8030	6200	7156	4856	4383
	**GP's**	Number of.	270	235	342	379	472	752	1033	1185
		Pop/GP's	6444	8132	6047	5678	4873	2988	2059	2030
**Region 16**	**Total phys.**	Total Physc	128	78	104	165	252	440	676	877
		Pop/Physc	5500	10872	9596	7176	5437	3445	2506	2213
	**Specialists**	Number of.	37	19	41	32	84	100	188	272
		Pop/Specia	19027	44632	24342	37000	16310	15160	9011	7136
	**GP's**	Number of.	91	59	63	133	168	340	488	605
		Pop/GP's	7736	14373	15841	8902	8155	4459	3471	3208

### Change of gini index

Gini coefficients that are calculated for the analysis of inequalities in the distribution of physicians are shown in Table 2. It also demonstrates a serious decrease in the unequal distribution of physicians between 1965 and 2000 in Turkey. In 1965, the Gini for total physician is quite high (0.47), and in 2000 it decreases considerably (0.20). In 1965, the Gini for GPs and specialists are 0.44 and 0.52, respectively and in 2000 these values decrease to 0.13 and 0.28, respectively. The inequality in the distribution of specialists is still at an important level.

**Table 2:** **Gini indices for three categories between 1965 and 2000**

**Years**	**Gini total**	**Gini GP**	**Gini specialist**
**1965**	0.47	0.44	0.52
**1966**	0.46	0.44	0.51
**1967**	0.45	0.44	0.49
**1968**	0.46	0.42	0.51
**1969**	0.47	0.48	0.49
**1970**	0.48	0.47	0.49
**1971**	0.49	0.48	0.50
**1972**	0.47	0.43	0.50
**1973**	0.46	0.47	0.46
**1974**	0.49	0.49	0.49
**1975**	0.49	0.48	0.47
**1976**	0.49	0.53	0.47
**1977**	0.45	0.45	0.47
**1978**	0.46	0.45	0.47
**1979**	0.44	0.39	0.49
**1980**	0.42	0.35	0.49
**1981**	0.42	0.37	0.47
**1982**	0.36	0.32	0.42
**1983**	0.37	0.29	0.44
**1984**	0.35	0.29	0.41
**1985**	0.34	0.26	0.41
**1986**	0.33	0.25	0.39
**1987**	0.33	0.27	0.40
**1988**	0.30	0.22	0.38
**1989**	0.27	0.18	0.36
**1990**	0.25	0.17	0.34
**1991**	0.25	0.17	0.35
**1992**	0.23	0.16	0.33
**1993**	0.24	0.17	0.33
**1994**	0.22	0.16	0.33
**1995**	0.22	0.16	0.31
**1996**	0.23	0.18	0.31
**1997**	0.22	0.17	0.31
**1998**	0.23	0.16	0.31
**1999**	0.20	0.14	0.29
**2000**	0.20	0.13	0.28

In the first period between the years 1965–1980, there is not a considerable amount of decrement in the Gini index compared to the second period between the years 1981–1995 during which a dramatic decline is observed (Table 3).

**Table 3:** **Changes in gini index**

**Years**	**Gini total**	**% Change**	**Gini GP’s**	**% Change**	**Gini specialists**	**% Change**
**1965**	0.47	-	0.44	-	0.52	-
**1970**	0.48	2.13	0.47	6.82	0.49	−5.77
**1975**	0.49	2.08	0.48	2.13	0.47	−4.08
**1980**	0.42	−14.29	0.35	−27.08	0.49	4.26
**1985**	0.34	−19.05	0.26	−25.71	0.41	−16.33
**1990**	0.25	−26.47	0.17	−34.62	0.34	−17.07
**1995**	0.22	−12.00	0.16	−5.88	0.31	−8.82
**2000**	0.20	−9.09	0.13	−18.75	0.28	−9.68

The geographic distribution of physicians was seriously unequal during the first period. Geographic disparities in physician density were still quite high at the beginning of 1980s. The Turkish authoritarian government at the beginning of 1980s passed the “compulsory service law” to improve the geographic distribution of physicians. At the same time the quotas for medical students were also increased. Despite these interventions, the inequality was still present in 2000, but it decreased.

Concentration of physicians in developed-urban regions is observed among both GP’s and specialists. The degree of this concentration is higher in specialists than in GP’s (Table 2). This tendency is driven during all years and two periods. But inequalities have been decreasing and this decrease is especially remarkable in the second period when the two years of compulsory service for newly appointed physicians and newly appointed specialists is enacted.

### Changes in mal-distribution and efficacy of regulation

**Figure 3:**
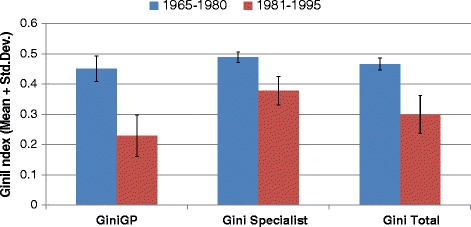
**Gini index by periods.**

For the total period, 1965–1995, it has been determined that the difference between the average Gini index of general practitioners (GPs) and specialists is significant (p < 0.01) (Tables 4 and 5). The average Gini index of GPs is lower than that of specialists, indicating that the geographic distribution among GPs is better (i.e. shows more equality) than specialists. As the Figure 3 suggests, the Gini coefficient for the GPs has almost always been lower than that of the specialists. In order to test whether the Gini coefficient for the GPs has statistically been lower than the Gini coefficient of the specialists, we conduct the test of equality of these two coefficients over time by using the standard Z-test. We find Z = 8.724 with p < 0.000, suggesting that the Gini coefficient for the GPs has indeed statistically been lower than the Gini coefficient of the specialists.

**Table 4:** **Period under discussion**

	**Count**	**Percentage**
**1965-1980**	16	51.61
**1981-1995**	15	48.39
**Total**	31	100

**Table 5:** **Gini scores by periods**

**Period**		**Gini total**	**Gini GP**	**Gini specialist**
**1965-1995**	**Mean**	0.385	0.344	0.435
**N = 31**	**Std. Dev**	0.096	0.125	0.066
	**Median**	0.42	0.37	0.47
	**Minimum**	0.22	0.16	0.31
	**Maximum**	0.49	0.53	0.52
**1965-1980**	**Mean**	0.466	0.451	0.488
**N = 16**	**Std. Dev.**	0.0200	0.042	0.017
	**Median**	0.465	0.45	0.49
	**Minimum**	0.42	0.35	0.46
	**Maximum**	0.49	0.53	0.52
**1981-1995**	**Mean**	0.299	0.229	0.378
**N = 15**	**Std. Dev.**	0.063	0.069	0.047
	**Median**	0.30	0.22	0.38
	**Minimum**	0.22	0.16	0.31
	**Maximum**	0.42	0.37	0.47
***P***		0.001**	0.001**	0.001**

Mann Whitney *U* test **p < 0.01.

It has been found that the difference between average Gini index of two periods is significant for both GPs and specialists. The average Gini index of the second period is lower than that of first period for both doctor groups (namely GPs and specialists). The significance of differentiation between first and second period is analysed through Mann–Whitney *U* test (p < 0.001). This means that the doctor distribution improved significantly within the second period; the result is consistent for both GPs and specialists.

### The analysis of improvement in gini index

**Figure 4:**
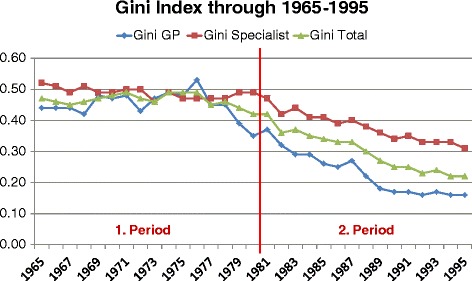
**Gini index for two periods.**

In the previous section, it is remarked that the average Gini index of both GPs and specialists is significantly lower in the second period compared to first period. The Gini index exhibits a downward trend through the years (Figure 4).

In order to confirm this trend and to determine how this trend changes among doctor groups and periods, regression analysis is used. Before estimating the regression equation, we test stationarity of the series. For this purpose, we apply the stationarity test proposed by Kwiatkowski et al. [18]. The results of this stationarity test are provided below in Table 6.

**Table 6:** **Stationarity test results**

**Series**	**Test statistic**
**Physicians (total)**	0.142
**GP**	0.114
**Specialists**	0.165

Notes: Test includes constant and trend. Critical value of the test statistic at 1% significance level is 0.216.

As the estimated test statistics for all three variables are less than the critical value, the null hypothesis of stationarity cannot be rejected at 1% significance level. This finding implies that all the three series under investigation are stationary, and hence, regression results will be robust. Therefore, we proceed to estimate the regression equations.

The linear regression model is applied to the data. “Gini index” is the dependent variable and the time is the independent variable. Initially, separate regression models (equations) for each doctor group focusing on the total period are formed (1965 – 1995). Later on, for each period and for each doctor group regression models have been set. Below one can find regression equations on which our model is based:

$$ \begin{array}{l}\begin{array}{l} Gini\_GP = a + b*Yea{r}_{1965-1995}\hfill \\ {} Gini\_ Specialist = a + b*Yea{r}_{1965-1995}\hfill \\ {} Gini\_GP = a + b*Yea{r}_{1965-1980}\hfill \\ {} Gini\_ Specialist = a + b*Yea{r}_{1965-1980}\hfill \\ {} Gini\_GP = a + b*Yea{r}_{1981-1995}\hfill \end{array}\\ {} Gini\_ Specialist = a + b*Yea{r}_{1981-1995}\end{array} $$

Results have been presented below (Table 7):

**Table 7:** **Regression analysis**

**Period**		**a (constant)**	**b**	Conf. interval of b*	**R** ^**2**^
**1965-1995**	**GP**	0.545	−0.013	−0.015/-0.010	0.832
**N = 31**	**Specialist**	0.544	−0.007	−0.008/-0.006	0.889
**1965-1980**	**GP**	0.465	0.002**	−0.007/0.003	0.036
**N = 16**	**Specialist**	0.509	−0.002	−0.004/-0.001	0.430
**1981-1995**	**GP**	0.578	−0.015	−0.017/-0.012	0.898
**N = 15**	**Specialist**	0.622	−0.010	−0.012/-0.009	0.941

*: 95% confidence level.

**: Statistically not significant.

Between 1965 and 1995 (total period), average decrease in Gini index is 0.013 (standard error is 0.001) per annum in GP doctor group. On the other hand, the average decrease in Gini index in specialist group is 0.007 (std error is <0.001). We can conclude that the rate of decrease in Gini index is significantly higher in GP group compared to specialist. For both regression model R^2^ is reported as above 0.80 indicating that linear regression model represents real situation well enough. That is to say, linear regression model fits the examined data.

Regression analysis with regard to two different periods reveals that in the first period (1965–1980) the regression model for the GP group is not significant (i.e. b = 0), meaning that we cannot conclude a linear trend for this period for GPs. In the specialist group a significant downward linear trend is noted, nevertheless the magnitude is small (b = −0.002; confidence interval −0.004/-0.001). However R^2^ (0.43) is lower than the required for a model to be representative of the real situation.

On the other hand, the regression analysis of the second period (1981–1995) reveals more conclusive results. The average decrease in Gini index per annum is −0.015 (std. error 0.001) for the GP group and 0.010 (0.001) for the specialist group. It can be clearly concluded that the rate of decrease in Gini index in the second period is significantly higher in the GP group compared to the specialist group. In other words, the rate of improvement in GP distribution is faster than that of specialists. Another consistent finding by Mann–Whitney U is shown at Table 8. According to results, there is a significant difference between GPs and specialists (p < 0.05).

**Table 8:** **Comparing of changes in rate of gini index decrease between GPs and specialists**

**Test statistics**	**Value**
**Mann-Whitney U**	8.000
**z**	−4.341
**Asymp. Sig. (2-tailed)**	.000
Exact Sig. [2*(1-tailed Sig.)]	.000 (a)

The following model is developed in order to analyse the effects of both the increasing number of physicians and the government regulation. A multiple regression analysis is conducted to estimate the model parameters.

$$ Gini={\beta}_0+{\beta}_1 Phsician\  per\ 10000\  people+{\beta}_2 Regulation+{\beta}_3 Time+\varepsilon $$

Table 9 shows the estimated results of the multiple regression equation.

**Table 9:** **Regression analysis results for estimated variables**

	**Coefficient**	**t-statistics**
Physician per 10000 people	−0.035*	−4.27
Regulation Dummy	−0.051*	−4.07
Time	0.001	0.41
Constant	0.619*	30.04
Adjusted-R^2^	0.935	
Prob > F	0.00	

Dependent Variable: Gini coefficient. *denotes 0.01 level of significance.

Overall model explains 93.5% of the variation in the Gini coefficient with three independent variables. The model is jointly significant at the 0.01 significance level. The regulations imposed by the government have a significant impact on the Gini. It indicates that the Gini coefficient decreased by 0.051 points when the law came into force. The effect of the *Physician per 10000 people* is also significant as expected. When the number of *Physician per 10000 people* increased by 1, the Gini coefficient decreased by 0.035 points.

## Discussion

Standard location theory assumes that free market mechanism does not fail about physician location behaviour. According to standard location theory, as the number of physicians increases, the diffusion of the physicians from the centre to the periphery will spontaneously occur associated with the decrease in their income [10]. “Standard economic theory (neoclassical) assumes that physicians seek to maximize their profit and therefore tend to practice in region with high income” [3]. But in reality, this is not probable under this assumption since the physicians would create their own demands. The ability of creating their own demands does provide autonomy about the location of physicians. This ability will also cause an increment of supply of health services and expenditures which will provide the resources to be directed to physicians.

Some authors assume that physicians maximize utility rather than profit [9]. Utility function includes non-economic quality of life factors (i.e., percent graduates and professionals located in the area, public school expenditures, non-public teachers per capita, and sufficient hospital beds etc.) [8]. Population, people with high income, big-sized general hospitals, special branch and university hospitals, social utilities have been concentrated in big, developed, metropolitan and seaside cities or areas. Therefore, assumption of standard theory must be built on “utility” concept; otherwise, the uneven distribution of physicians must be accepted as a display of market failure.

Naturally, the concentration tendency of physicians in these urban-developed areas cannot be avoided. Most of the studies done in several countries have indicated that despite the increase in the number of physicians, the overall uneven geographic distribution has not decreased [3]. The number of physicians in non-metropolitan counties and rural areas increases more slowly than that in metropolitan and urban areas. Even though the number of physicians increases, the unequal distribution of physicians could not be improved adequately or the number of physicians in rural regions increases rather slowly when compared with the ratio in metropolises and urban regions. In the literature, it has been reported that despite the relative increase in the number of physicians in proportion to the population, the inequality in the distribution of physicians did not diminish, and increased at that [19,20].

The ratio of population to physicians is decreased spontaneously when the growth rate of physician number is bigger than the population growth rate. But this momentum of decrease is not same for the developed-urban and the undeveloped-rural areas. Physicians will not diffuse to all cities/regions with the same proportion as their numbers increase. Developed regions or urban cities will absorb newly graduated physicians because of the physician shortage and increasing demand for new medical services. Without government intervention, physicians would prefer attractive cities/regions and as a result of these preferences, there would be an uneven geographic distribution of physicians [5]. The situation of Turkey before the start of compulsory service practice in 1980, namely the rate of inequality in the number of physicians which almost remained the same even when the number of doctors arose is consistent with this.

The inequality in the distribution of physicians is higher for specialists than GPs [11]. Especially “specialists will serve comparatively larger market areas than family practitioners and general practitioners” [10]. The inequality in the distribution of specialists who are under the effect of market motivations (profit maximizing) is more significant. For example, Fülöp et al. [5] found that the regional distribution disparity is less pronounced in Germany than in Austria but also differences can be seen most clearly for specialists in both countries (Gini coefficients are significantly higher for specialists to general practitioners in both countries). Meliala et al. [21] found that there is substantial inequality in the distribution of specialist doctors in Indonesia. It is also likely that there is a concentration of specialist doctors in urban areas, where most hospitals are located. Moreover, the fact that they earn a rather high salary in cities due to private work practice is another factor behind this concentration. The outcomes of this study are consistent with these results. For all years (35 years) analysed, the Gini index for specialists, which is a measurement of inequality, is higher than the GPs index.

The health system of a country is deemed to be effective by looking at the distribution of primary care physicians [4]. In Turkey, primary health care services are mainly provided by GPs. Thus, the distribution of GPs is the most important variable of the primary care. Together with the regulation about compulsory service, a significant decrement has been observed for the Gini index of both groups -specialists and GPs- where it was more dramatic for GPs. Similarly, Matsumoto et al. [4] found that the distribution of primary care physicians in Britain is more equitable than in Japan since it is better regulated in Britain.

Newhouse et al. shows that, as the supply of physicians grow, medical and surgical specialists diffuse into smaller communities in the United States. “Contrary to conventional wisdom, physicians will diffuse to nonmetropolitan areas in response to growth in supply” [10]. Other evidence suggests that increasing the number of physicians has only a small impact on reducing the disparities seen in their geographical distribution [3]. For example, an increase in the number of physicians in Japan from 1980 to 1990 did not improve the inequality in physician distribution [22]. Sasaki et al. [23] find that more urbanized regions have more pediatricians and the total increase in pediatricians during 2002–2007 was primarily absorbed into the urban areas.

Increase in the supply of physicians in Turkey does not have a sizable effect –only a small effect, Gini index decreases from 0.47 to 0.42 between 1965 and 1981- on improving the geographic distribution of physicians up to the beginning of the 1980s. Newly graduated physicians do not go to the rural and nonmetropolitan areas even though real income in these areas is higher.

However, there is a dramatic decrement in the Gini index between 1981 and 1995 due to the compulsory service law. And also in the same period, the quotas for medical students have been increased, thus providing a positive effect for this decrement.

It can be argued that the Gini coefficient has declined as a result of increase in number of physicians during the analysed period, and hence, the regulation had a limited effect on reduction in the Gini coefficient. Our finding suggests that, the regulation in fact lowered the Gini coefficient in Turkey, and this decrease was statistically significant. While the improvement in the 1^st^ period (a small decrement in the Gini index from 0.47 to 0.42) does only depend on to the increment in the physician number, the majority of the improvement (decrement in the Gini index from 0.42 to 0.22) in the 2^nd^ period does mainly depend on the regulation.

In the research carried out by Yardım and Üner with respect to the unequal distribution of physicians in Turkey, the value of Gini for total physician for the year of 2010 was calculated as 0,14 [24].

## Conclusions

One of the main weaknesses of the health system in Turkey is that there has not been an optimal distribution of physicians. In this study, the changes in the inequality of the physician distribution is analysed for Turkey by considering 16 regions and 35 years. In the early years of the health policy, the increase in the number of medical practitioners is the primary target while the government intervention in the physician distribution receives much less attention. The improvement of the physician distribution is one of the main objectives between the years 1980 and 2000. The increment of the physician supply is an important factor in reducing the inequalities in the physician distribution. This improvement is especially obvious between 1981 and 1995 when the government introduced a strict two-year compulsory service for newly graduated both GP’s and specialists.

As a result, it is observed that the inequalities in the distribution between GPs and specialists are significantly different; inequality of specialist distribution is higher than the GP. The government intervention in the second period (1981–1995) provides an effective and fast improvement in the physician distribution. The decrement in the inequality for GP distribution is seen to be in higher ratios than the specialist. In other words, the rate of improvement in GP distribution is faster than that of specialist.

The findings indicate that the improvement of physician distribution lasts too long when it is left to market mechanism or it does not develop adequately. This phenomenon is more dominant for specialists under market motivation effect than it is for GPs.

## Endnote

^a^See: Jiang, H.J. and Begun, J.W. [2] for an ecological perspective.
